# Simultaneous activation and inhibition of autophagy sensitizes cancer cells to chemotherapy

**DOI:** 10.18632/oncotarget.10873

**Published:** 2016-07-28

**Authors:** Kwan-Hwa Chi, Yu-Shan Wang, Yi-Chun Huang, Hsin-Chien Chiang, Mau-Shin Chi, Chau-Hwa Chi, Hsin-Ell Wang, Shang-Jyh Kao

**Affiliations:** ^1^ Department of Radiation Therapy and Oncology, Shin Kong Wu Ho-Su Memorial Hospital, Taipei, Taiwan; ^2^ Department of Biomedical Imaging and Radiological Sciences, National Yang-Ming University, Taipei, Taiwan; ^3^ Department of Research and Development, JohnPro Biotech Inc., Taipei, Taiwan; ^4^ Institute of Veterinary Clinical Science, National Taiwan University, Taipei, Taiwan; ^5^ Division of Pulmonary Medicine, Department of Internal Medicine, Shin Kong Wu Ho-Su Memorial Hospital, Taipei, Taiwan

**Keywords:** autophagy, rapamycin, chloroquine, chemosensitization, synthetic lethality

## Abstract

While combined chemotherapy (CT) with an autophagy inducer and an autophagy inhibitor appears paradoxical, it may provide a more effective perturbation of autophagy pathways. We used two dissimilar cell lines to test the hypothesis that autophagy is the common denominator of cell fate after CT. HA22T cells are characterized by CT-induced apoptosis and use autophagy to prevent cell death, while Huh7.5.1 cells exhibit sustained autophagic morphology after CT. Combined CT and rapamycin treatment resulted in a better combination index (CI) in Huh7.5.1 cells than combined CT and chloroquine, while the reverse was true in HA22T cells. The combination of 3 drugs (triplet drug treatment) had the best CI. After triplet drug treatment, HA22T cells switched from protective autophagy to mitochondrial membrane permeabilization and endoplasmic reticulum stress response-induced apoptosis, while Huh7.5.1 cells intensified autophagic lethality. Most importantly, both cell lines showed activation of Akt after CT, while the triplet combination blocked Akt activation through inhibition of phospholipid lipase D activity. This novel finding warrants further investigation as a broad chemosensitization strategy.

## INTRODUCTION

Resistance to chemotherapeutics has become a major obstacle in successful cancer treatment, and there is an urgent need to develop novel treatment strategies [1]. Autophagy is a lysosome-mediated catabolic process, which aids in maintaining cellular homeostasis and survival during exposure to extra- or intracellular stresses through the degradation of targeted cytoplasmic components [2, 3]. Autophagy is usually cytoprotective when a cell experiences starvation or chemotherapy (CT) [4–6]. However, autophagy can switch from a cytoprotective to a cytotoxic role, depending on the extent of autophagy, autophagy flux, and apoptosis competence [7–9]. Apoptosis events are usually preceded by autophagy-dependent survival signals, while inhibition of autophagy usually promotes apoptotic cell death [10]. Chloroquine (CQ), a commonly used autophagy inhibitor, has been shown to reverse CT resistance in cultured cells, animal models, and patients [11]. However, it is still controversial whether CQ enhances the cytotoxic effects of everolimus [12, 13]. It is also not clear whether autophagy promotes or inhibits drug sensitivity. Current data suggest that mTOR inhibition by everolimus should increase the efficacy of CT or hormone therapy in breast cancers [14, 15].

While cell lethality signals may go through an autophagy process, “autophagic cell death” might be a misnomer [16]. Autophagy can be activated as a response to various stressors including CT. A completed autophagy process includes autophagosome formation, followed by autophagolysosome formation and degradation of autophagolysosome content. A proficient autophagy cell line may sustain autophagic morphology to prevent apoptosis. Autophagy has been regarded as a mediator of CT-induced cell death [17]. Inhibition of autophagolysosome formation by CQ may activate the unfolded protein response (UPR), an alternative way of dealing with stress; or result in autophagosome accumulation and necrotic death without UPR. The inhibition of autophagy by CQ may induce a persistent endoplasmic reticulum (ER) response and subsequent C/EBP homologous protein (CHOP) expression, which is potentially cytotoxic [18].

Both cytoprotective and cytotoxic autophagy can activate the upstream phosphatidylinositol 3-kinase (PI3K)/protein kinase B (Akt)/mechanistic target of rapamycin (mTOR) pathway to promote survival. CT may activate Akt via second messenger pathways initiated by hydrolysed membrane phospholipids [19]. Recent reports have suggested a link between phospholipase D (PLD) and Akt activity in a variety of cancers [20, 21]. Simultaneous inhibition of the mTOR pathway and suppression of the PI3K/Akt survival pathway during CT elicits a stronger inhibition of cancer cells than CT alone [22].

The interesting theory of “battery operated tumor growth” proposes that cancer cells induce an autophagy state in the tumor microenvironment leading to the increased production of recycled stromal nutrients to fuel the anabolic cancer cells [23, 24]. A synergistic interaction of autophagy inducers and inhibitors has been termed as the “autophagy paradox” [25]. In a pilot study, we have successfully tested the idea that triplet combination causes sequential hits to cancers [26]. Here, we investigate the underlying mechanisms of rapamycin (Rapa) and CQ treatment under conditions of high ER stress induced by vinca alkaloid [27] in both apoptosis-competent and apoptosis resistant hepatoma cells. We conclude that triplet drug combination not only inhibits the mTOR pathway but also suppresses the activation of Akt pro-survival signals more than any of the doublet treatment combinations (Rapa+V, CQ+V, and Rapa+CQ). Since both drugs are clinically available and cheap, this triplet treatment strategy may have broad clinical applicability.

## RESULTS

### Triplet drug combination was more cytotoxic than doublet drug combinations

**Figure 1:**
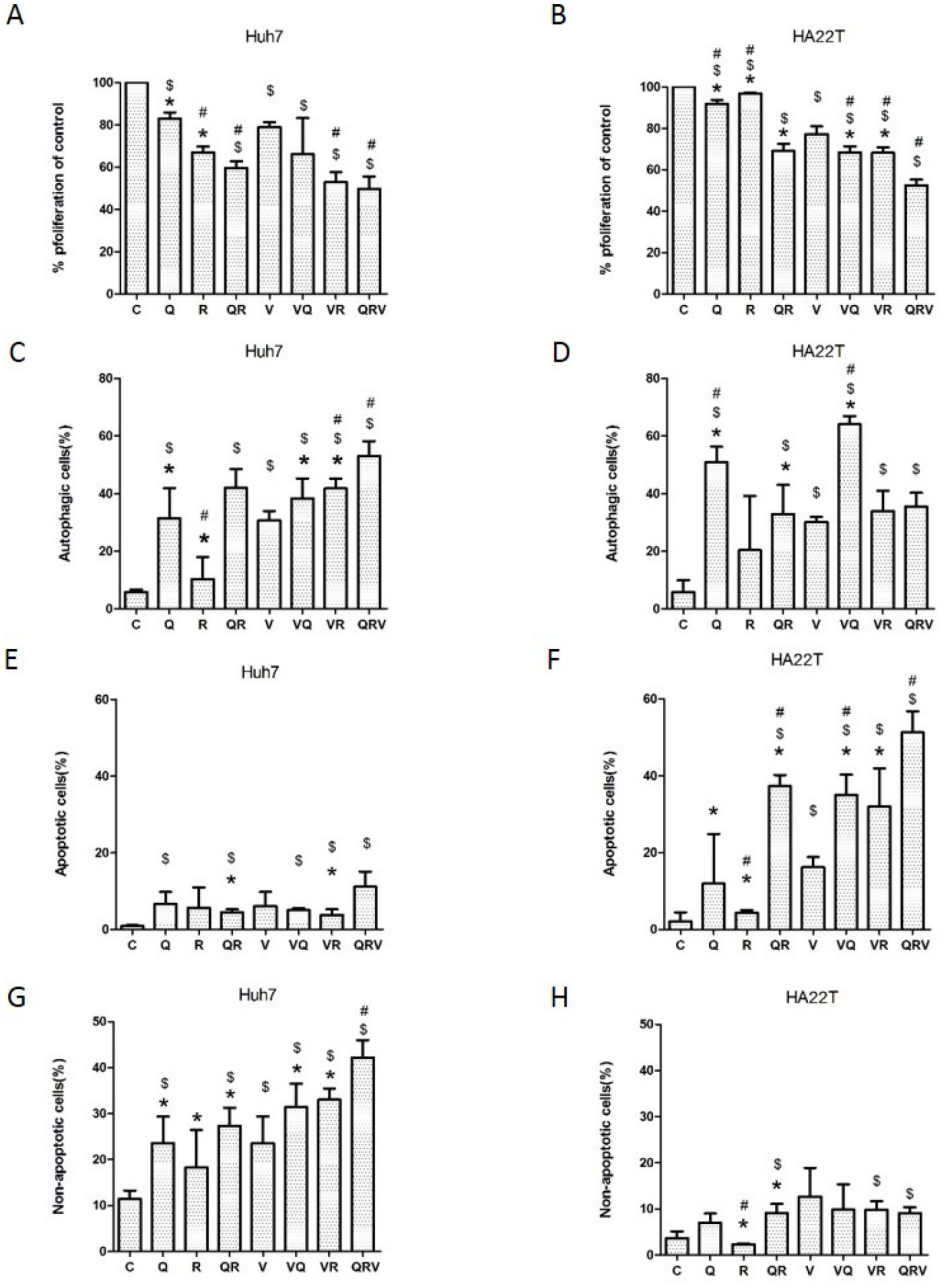
Cell proliferation, formation of acidic vesicular organelles (AVO), and induction of Annexin-V positive cells after combination drug treatment Cell proliferation in Huh7.5.1 **(A)** and HA22T **(B)** cells treated with CT drugs, with or without CQ, Rapa or CQ and Rapa, for 48 h, was analyzed using MTS. The formation of AVO was quantified by flow cytometry after acridine orange staining in Huh7.5.1 **(C)** and HA22T **(D)** cells that underwent the same treatments as in (A) and (B). The autophagic cells are presented as the percentage of acridine orange stained cells with positive fluorescein intensity. Flow cytometry was used to assess apoptotic and non-apoptotic cell death in Huh7.5.1 **(E** and **G)** or HA22T **(F** and **H)** cells. Huh7.5.1 and HA22T cells were treated as in (A) and (B). Apoptosis was measured using flow cytometry after staining with FITC-conjugated Annexin V and propidium iodide (PI). Positively stained cells were counted using FACSCalibur. Apoptotic cells were Annexin V positive and non-apoptotic cells were Annexin V negative and PI positive. Data are presented as mean ± SD of 3 experiments. Symbols indicate statistically significant differences in comparison to different treatments: Compared with control: $ = P < 0.05, Compared with vinorelbine:# = P < 0.05, Compared with CQ+Rapa+V: * = P < 0.05, via 2-tailed Student's *t* test.

Cell proliferation in two hepatoma cell lines, Huh7.5.1 and HA22T, was evaluated after triplet combination or doublet combination treatment. Several ratios of CT agents, CQ, and Rapa were tested in order to establish the optimal molar ratio of these three drugs. Combination indices (CI) were calculated as described in the Materials and Methods. The combination of CQ and Rapa at a molar ratio of 3:1 produced a synergistic effect at the level of 30–70% inhibition of cell proliferation in HA22T cells, with CI values of 0.661–0.976 and slight antagonism (CI = 1.331) at 90% inhibition (Table 1). In Huh7.5.1 cells, the CQ and Rapa combination at a molar ratio of 3:1 had an additive effect (CI = 0.998) at 30% inhibition and antagonism and CI values of 1.16, 1.47, and 2.49 at 50%, 70%, and 90% inhibition, respectively. Three-drug combinations of CQ, Rapa, and either vinorelbine (6:2:1), docetaxel (6:2:1), cisplatin (3:1:1.6), 5-FU (3:1:500), or gemcitabine (3:1:500) were tested. Greater cell growth suppression was achieved by the triplet combination compared with the doublet combinations. CIs suggested a synergistic effect at the level of 30–70% inhibition for treatment with CQ and Rapa, plus vinorelbine or docetaxel, in HA22T cells; a similar effect was observed for Huh7.5.1 cells (Table 1). The combination of CQ, Rapa, and vinorelbine produced the highest synergism and was selected for further experiments (Table 1). As shown in Figure 1A&1B, the triplet drug combination inhibited cell proliferation in both cell lines (P < 0.05).

**Table 1:** Combination index for combination drug treatment in Huh7.5.1 and HA22T cell lines

Huh7 cell line Drug combination	CI Values at % inhibition			
	30	50	70	90
CQ+Rapa (3:1)	**0.999**	1.160	1.467	2.493
(CQ+Rapa)+V (3:1:0.5)	**0.446**	**0.627**	**0.924**	1.785
(CQ+Rapa)+Tax (3:1:0.5)	**0.444**	**0.653**	1.034	2.154
(CQ+Rapa)+Cis (3:1:1.6)	1.214	1.169	1.144	1.153
(CQ+Rapa)+5-FU (3:1:500)	4.932	0.539	1.092	5.097
(CQ+Rapa)+Gem (3:1:500)	**0.794**	**0.876**	**0.973**	1.163

HA22T cell line Drug combination	CI Values at % inhibition			
	30	50	70	90
CQ+Rapa (3:1)	**0.661**	**0.803**	**0.976**	1.331
(CQ+Rapa)+V (3:1:1)	**0.302**	**0.459**	**0.875**	2.805
(CQ+Rapa)+Tax (3:1:1)	**0.443**	**0.567**	**0.732**	1.101
(CQ+Rapa)+Cis (3:1:3.2)	**0.572**	**0.755**	1.03	1.741
(CQ+Rapa)+5-FU (3:1:200)	**0.46**	**0.804**	1.435	3.619
(CQ+Rapa)+Gem (3:1:400)	**0.405**	1.056	2.964	15.784

V, Vinorelbine; Tax, Taxotere; Cis, Cisplatin; Gem, Gemzar. The ranges CI < 1, CI = 1, and CI > 1 indicate synergism, additive effect, and antagonism, respectively.

### Triplet drug combination promoted autophagy in Huh7.5.1 cells and apoptosis in HA22T cells

Because Rapa induces autophagy and CQ inhibits autophagolysome formation, we examined how the triplet drug combination affected patterns of cell death. Triplet drug combination treatment elevated the level of autophagy in comparison to the doublet combinations (Rapa+V, CQ+V, or Rapa+CQ) in Huh7.5.1 cells (Figure 1C), and eventually induced marked autophagy and non-apoptotic cell death (Figure 1C&1G). In HA22T cells, although CQ alone and doublet combinations (Rapa+V, CQ+V, or Rapa+CQ) induced autophagy (Figure 1D), they did not cause major cell death (Figure 1H). All doublet combinations (Rapa+V, CQ+V, or Rapa+CQ) as well as the triplet combination (Rapa+CQ+V) increased apoptotic cell death in HA22T cells (Figure 1F). These results indicate that co-administration of CQ and Rapa enhances chemo-sensitivity in both cell lines, regardless of whether it induces apoptosis or autophagy.

**Figure 2:**
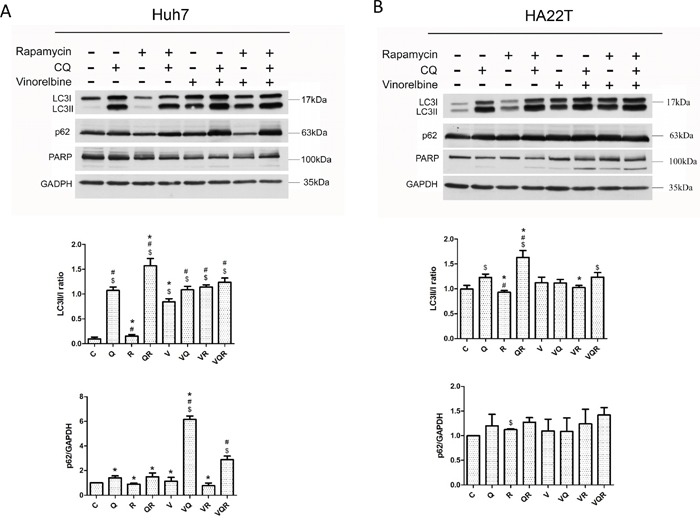
Western blot analysis of autophagy markers LC3II and p62 and apoptosis marker PARP in hepatoma cells after combination drug treatment Huh7.5.1 **(A)** and HA22T **(B)** cells were treated with vinorelbine, with or without CQ, Rapa or CQ and Rapa. After incubating 48 h, cells were harvested for western blot analysis. GAPDH was used as an internal control. Symbols indicate statistically significant differences in comparison to different treatments: Compared with control: $ = P < 0.05, Compared with vinorelbine:# = P < 0.05, Compared with CQ+Rapa+V: * = P < 0.05, via 2-tailed Student's *t* test.

An efficient autophagy process includes autophagosome formation and lysosome removal. Both cell lines responded differently to vinorelbine, which induced cytotoxic autophagy in Huh7.5.1 cells and cytoprotective autophagy from HA22T cells. Huh7.5.1 cells are characterized by high autophagy flux and proficient autophagy activity as indicated by no basal microtubule-associated protein 1A/1B-light chain 3-phosphatidylethanolamine conjugate (LC3II) signal, a low LC3II/cytosolic LC3 (LC3I) ratio, low nucleoporin 62 (p62) accumulation after mTOR inhibition by Rapa, and accumulation of LC3II and p62 after lysosome inhibition by CQ. In contrast, HA22T cells have less autophagy flux as indicated by higher LC3II and p62 accumulation after Rapa treatment (Figure 2A&2B). In HA22T cells, triplet combination increased autophagy vesicular formation without causing a switch to apoptosis. HA22T cells are more apoptosis-prone, thus PARP cleavage occurred in HA22T cells after either doublet or triplet treatment. Only mild PARP cleavage of Huh7.5.1 cells was seen after triplet treatment.

### Triplet drug combination reduced activation of Akt through decreased PLD activity

**Figure 3:**
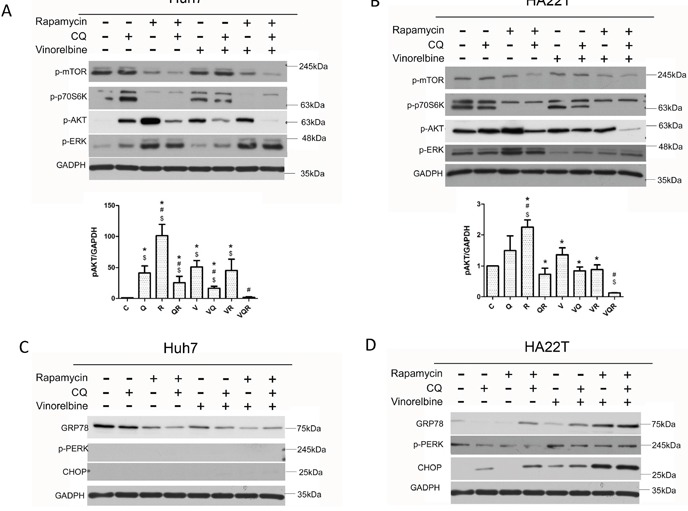
Impact of combination drug treatment on cell signaling pathways Huh7.5.1 **(A, C)** and HA22T **(B, D)** cells were treated with vinorelbine, with or without CQ, Rapa, or CQ and Rapa. After incubating 48 h, cells were harvested for western blot analysis to evaluate mTOR-Akt and ERK1/2 signaling **(A and B)**, ER stress response **(C and D)** and GAPDH was used as an internal control. Symbols indicate statistically significant differences in comparison to different treatments: Compared with control: $ = P < 0.05, Compared with vinorelbine:# = P < 0.05, Compared with CQ+Rapa+V: * = P < 0.05, via 2-tailed Student's *t* test.

**Figure 4:**
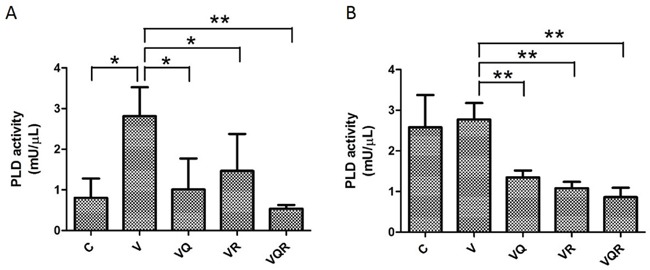
PLD activity after combination drug treatment PLD activity of Huh7.5.1 **(A)** and HA22T **(B)** cells treated with vinorelbine, with or without CQ, Rapa, or CQ and Rapa, for 48 h. Data are presented as mean ± SD of three experiments. Asterisks indicate statistically significant differences in comparison to untreated cells (* =*P* < 0.05, **= *P* < 0.01), via 2-tailed Student's *t* test.

The PI3K-Akt-mTOR pathway plays a pivotal role in apoptosis/survival signaling and is involved in chemo-resistance [28]. Phosphorylated mTOR and its downstream target kinase p70S6K were inhibited in both cell lines after Rapa treatment. However, both cells displayed feedback activation of phosphorylated Akt after Rapa treatment with or without CT. Most importantly, both cells had decreased levels of phosphorylated Akt after triplet drug treatment (Figure 3A&3B). Huh7.5.1 cells also had Ras/Raf/extracellular signal-regulated kinase (ERK) 1/2 activation after Rapa treatment (Figure 3A). Sustained activation of ERK has been shown to promote the death of many cancer cell lines [29]. Nevertheless, HA22T cells had decreased ERK activation after CT (Figure 3B). Instead, they had a strong and sustained ER stress response, as evident by increased of GRP78 and CHOP expression after triplet drug treatment. Huh7.5.1 cells showed no signs of an ER stress response (Figure 3C&3D). These results show that simultaneous inhibition of mTOR and Akt by the triplet drug combination treatment overcomes chemo-resistance. It has been reported that PLD activity is closely associated with Akt activation [21]. Triplet combination reduced PLD activity in both cell lines (Figure 4A&4B).

### Basal metabolic phenotypes and adaptive metabolic responses after triplet drug combination treatment

**Figure 5:**
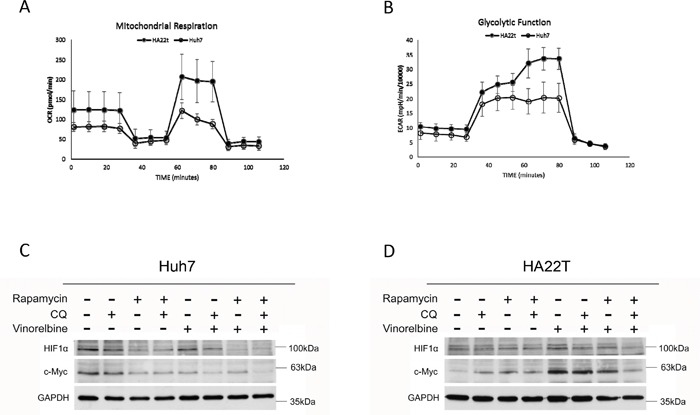
Basal metabolic phenotypes and mitochondrial damage after triplet drug combination treatment Basal metabolic phenotypes of Huh7.5.1 and HA22T cells were evaluated for OCR **(A)** and ECAR **(B)** with an Extracellular Flux Analyzer. Huh7.5.1 **(C)** and HA22T **(D)** cells were treated with vinorelbine, with or without CQ, Rapa, or CQ and Rapa. After incubating 48 h, cells were harvested for western blot analysis to evaluate energy metabolism, and GAPDH was used as an internal control.

To determine basal glycolytic activity and glycolytic capacity, we measured extracelluar acidification rate (ECAR) by consecutive injections of glucose, oligomycin, and 2-Deoxy-D-glucose (2-DG). Glycolytic activity and capacity were 10% and 40% higher in HA22T cells than Huh7.5.1 cells, respectively. The basal oxygen consumption rate (OCR) and OCR capacity were 35% and 60% lower in Huh7.5.1 cells as compared with HA22T cells (Figure 5A&5B). This suggested that the Huh7.5.1 cells are more dependent on glycolysis and have a more efficient autophagy process to recycle energy while HA22T cells adopt a more oxidative metabolism. The addition of Rapa and CQ decreased basal ECAR and OCR, as well as both capacities in both cells (data not shown).

**Figure 6:**
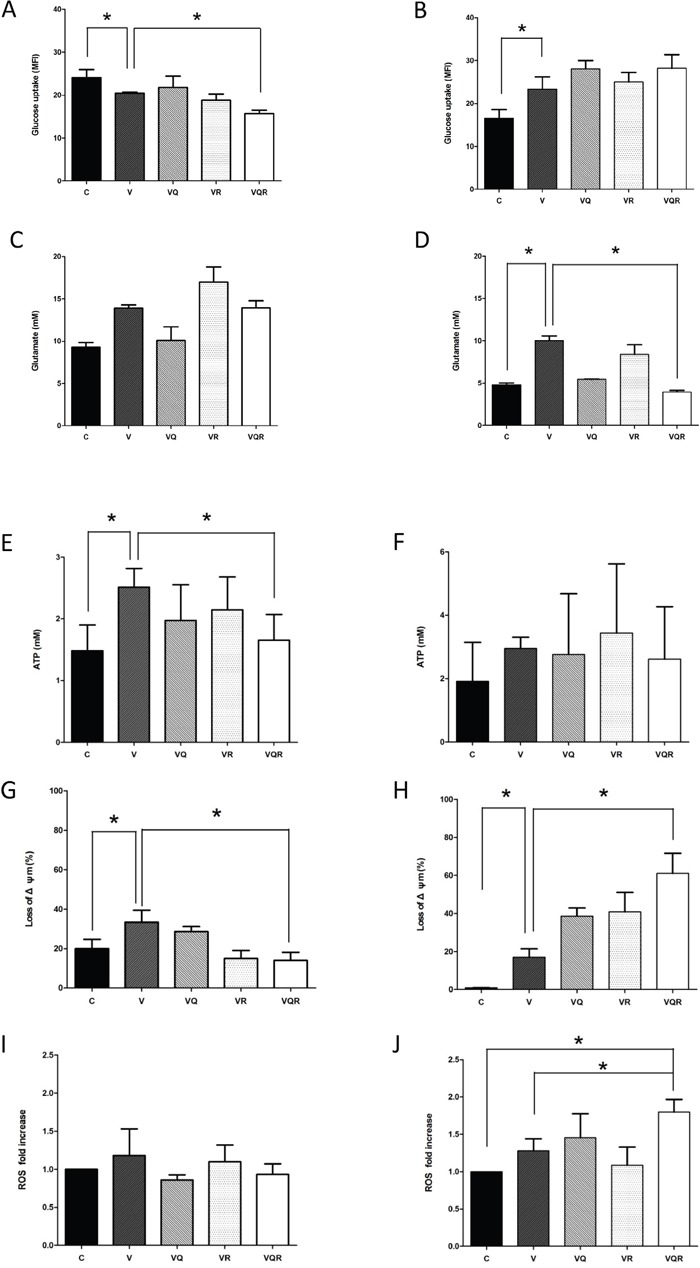
ATP generation, loss of mitochondrial membrane potential, glucose uptake, glutamine uptake, and ROS levels after combination drug treatment Glucose uptake for Huh7.5.1 **(A)** and HA22T **(B)** cells treated with vinorelbine, with or without CQ, Rapa, or CQ and Rapa, for 48 h was assayed using FACS tracings for NBD-2-deoxy-glucose. Intracellular glutamate production for Huh7.5.1 **(C)** and HA22T **(D)** cells was also investigated. Generation of ATP for Huh7.5.1 **(E)** and HA22T **(F)** cells, reduction in mitochondrial membrane potential (Δψm) for Huh7.5.1 **(G)** and HA22T **(H)** cells, and reactive oxygen species (ROS) levels for Huh7.5.1 **(I)** and HA22T **(J)** cells treated with vinorelbine, with or without CQ, Rapa, or CQ and Rapa, for 48 h. Data represent mean ± SD of three experiments. Asterisks indicate statistically significant differences in comparison to untreated cells (*P* < 0.05), via 2-tailed Student's *t* test.

Hypoxia inducible factor 1-alpha (HIF-1α) regulates genes involved in glycolysis [30] and the proto-oncogene c-Myc stimulates glutamine catabolism [31]. As shown in Figure 5C&5D, HIF-1α was inhibited in Huh7.5.1 cells after treatment with the triplet drug combination. HA22T cells had increased c-Myc expression after vinorelbine treatment and decreased expression after triplet drug treatment. HA22T cells are prone to compensatory over-expression of c-Myc and increase both glycolysis and mitochondrial activity after CT as an alternative energy source; however, c-Myc expression was decreased after triplet drug treatment. Dysfunctional autophagy impairs glutamine metabolism and results in reduced intracellular glutamate, decreased antioxidant intermediates, loss of mitochondrial membrane permeabilization (MMP), and increased reactive oxygen species (ROS) production from progressive mitochondrial damage [32, 33]. Glutamate may activate mitochondrial matrix configuration and decrease the loss of MMP [34]. Triplet drug treatment decreased glucose uptake in Huh7.5.1 cells (Figure 6A), intracellular glutamate levels in HA22T cells (Figure 6D), and ATP in Huh7.5.1 cells (Figure 6E) as compared with CT treatment alone. No difference in ATP was found in HA22T cells (Figure 6F), but marked loss of MMP (Figure 6H) and ROS damage (Figure 6J) were observed.

### Triplet drug combination inhibited the growth of hepatoma xenografts

**Figure 7:**
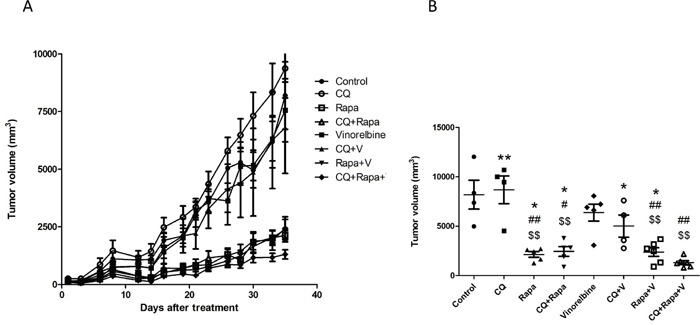
CQ and Rapa enhance the growth inhibition effect of vinorelbine *in vivo* The *in vivo* efficacy of CQ, Rapa, and vinorelbine alone, and in combination, was evaluated in a Huh7.5.1 xenograft model. **(A)** Male NOD/SCID mice were subcutaneously injected with 5 × 10^6^ cells per mouse in the right flank to induce tumor xenografts. Vinorelbine at a dose of 5 mg/kg was administered weekly for three weeks. Rapa at a dose of 5 mg/kg was administered five times per week, with two days off, for three cycles. CQ at a dose of 50 mg/kg was administered daily for three weeks. Groups consisted of a saline control (control), CQ, Rapa, CQ amd Rapa, vinorelbine, CQ and vinorelbine, Rapa and vinorelbine, and the triplet drug combination (vinorelbine, CQ and Rapa). Tumor size was evaluated by measuring the longest (a) and widest perpendicular (b) dimensions using a caliper and is expressed as a volume according to the formula 1/2a^2^b (mm^3^). **(B)** Average tumor size on day 35 after treatment. (Compared with control: $ = P < 0.05; $$ =P < 0.01, Compared with vinorelbine:# = P < 0.05; ## =P < 0.01, Compared with CQ+Rapa+V: * = P < 0.05; ** =P < 0.01; *** = P < 0.001), via 2-tailed Student's *t* test.

We next investigated whether double modulation of autophagy by CQ and Rapa is synergistic with chemotherapy in a hepatoma xenograft model. We failed to establish the HA22T hepatoma xenograft model in NOD/SCID mice, but Huh7.5.1 cells were successfully established. When tumor size reached 100 mm^3^, we started intraperitoneal injections of CQ (50 mg/kg daily for 5 days), Rapa (50 mg/kg daily for 5 days), and vinorelbine (5 mg/kg weekly), and made measurements of tumor sizes every fourth day (6 tumors/group). As shown in Figure 7, the triplet drug combination treatment (Rapa+CQ+V) more effectively inhibited tumor growth in the Huh7.5.1 xenograft than the doublet combination (Rapa+V, CQ+V or Rapa+CQ).

## DISCUSSION

In the current study, we have demonstrated that simultaneous activation and inhibition of autophagy by CQ and Rapa combination treatment is a novel therapeutic strategy for CT sensitization. The chemosensitization effect was achieved in both apoptosis-competent (HA22T) and apoptosis resistant (Huh7.5.1) cells. Increasing autophagy through CT and mTOR inhibition by Rapa may or may not induce additive effects. However, CQ combined with Rapa and CT, may either switch cytoprotective autophagy to apoptosis or sustain autophagy. We also found that the triplet drug combination overcame CT resistance by simultaneously decreasing mTOR and Akt.

The combined induction and inhibition of autophagy to activate cell death is an interesting idea in both cancer cell and cancer-cancer associated fibroblast models [25]. Seront et al. reported a robust antitumor effect with Rapa and CQ treatment for large hypoxic tumors, but not small tumors *in vivo* [35]. Whether autophagy plays a pro- or antitumorigenesis role depends on tissue type and tumor developmental stage [36]. Autophagy usually plays a pro-survival role in established tumors because it acts as a mechanism to overcome the stress associated with oncogenesis [37]. However, tumors with proficient autophagy activity typically succumb to mTOR inhibition. As seen in Figure 2A, Rapa treatment did not result in accumulation of LC3II and p62 in Huh7.5.1 cells, but CQ treatment caused high autophagy flux. High autophagy flux, as reflected by high accumulation of LC3II and p62 after CQ treatment, is usually regarded as a poor prognostic factor and is associated with cancer progression [38, 39].

The HA22T cells adopted a more mitochondria oxidative metabolism and Huh7.5.1 a more glycolytic metabolism. Triplet drug treatment decreased HIF-1α in Huh7.5.1 cells and subsequently decreased glucose uptake. This result suggests that the triplet drug combination is autophagy-dependent. Huh7.5.1 cells had less susceptibility to apoptosis but inefficient mitochondria, which resulted in necroptosis. In contrast, triplet drug combination also worked in apoptosis-competent cells like HA22T, where protective autophagy may be switched to mitochondria related apoptosis. c-Myc was increased in HA22T cells after CT as a mechanism of drug resistance. Hyperactive c-Myc activity orchestrates cells' response to metabolic demands including glucose and glutamine uptake [40]. Glutamine is critical for mitochondrial respiration [41] and depletion of glutamine or glutaminolysis inhibition triggers an apoptotic response in cells overexpressing c-Myc [42]. In c-Myc-inducible cells, CT triggers glutamine dependence. Interestingly, CQ has been reported to preferentially enhance the death of c-Myc over-expressing cells [43], and is a potent inhibitor of glutamate dehydrogenase [44].

We found that Huh7.5.1 cells had a high basal expression of chaperone protein GRP78, which inhibits apoptosis and stimulates pro-survival autophagy [45]. The high basal GRP78 expression coincided with proficient autophagy activity. Rapa treatment accelerated the autophagic process with a decrease of GRP78 expression. The addition of CQ induces defective autophagolysomal formation and aborted autophagy may increase cell death by necroptosis through p62 accumulation [46]. Interestingly, no ER stress response was noted after doublet or triplet treatment in this particular cell. Since ER homeostasis can be maintained without UPR response (p-PERK, CHOP activation), sustained autophagy without apoptosis was observed in Huh7.5.1 cells after triplet treatment. Thus, chaperone-mediated ER stress could be released by secretion of misfolded proteins outside of cells after triplet treatment without activation of ER associated protein degradation [47, 48].

In contrast, HA22T cells are apoptosis-competent and CT induced both apoptosis and autophagy in these cells. The addition of CQ to CT switched autophagy into apoptosis through ER-mitochondria cross-talk after autophagy blockage. Triplet drug treatment in HA22T cells increased CHOP, loss of MMP, and apoptosis without ERK activation. ER stress alone was not sufficient to induce apoptosis, but simultaneous inhibition of GRP78-dependent autophagy and canonical autophagy by CQ led to ER-induced apoptosis [49]. The triplet drug treatment caused higher levels of apoptosis than any of the doublet combinations. Inhibition of mTOR increases autophagy and abrogates its inhibitory effect on the PI3K/Akt pathway, which in turn leads to activation of Akt and increases cell survival [50]. CT with or without Rapa resulted in Akt activation, but only the triplet combination inhibited both mTOR and Akt activation. It has been recently reported that CQ exerts its anti-cancer effects partially through modification of the PI3K/Akt/mTOR pathway and overrides Rapa-induced Akt-phosphorylation [51]. PLD hydrolyzes membrane phospholipids to generate phosphatidic acid (PA). In turn, PA activates Akt to mediate survival signals [21]. CQ has been reported to inhibit PLD activity [52]. Triplet drug combination decreased PLD activity, which might be the most important rationale for the addition of CQ and Rapa to a CT regimen.

Both cell lines had autophagy as a common denominator but different stress response scenarios. Sustained autophagy reaching capacity (by CT and Rapa) followed by a blockage (by CQ) can cause toxic protein aggregates to form. It is a strategy of synthetic lethality that works in consecutive steps. The decrease of survival signals results in either necrosis or in mitochondrial damage through complex cross-talk among lysosomes, mitochondria, and the ER [53]. Cell death is a highly dynamic response that may manifest as a spectrum of morphological overlaps [54], and is an area of intense research. Rapa and CQ, apart from CT, exert only a limited cytotoxic effect [55–57]. Although it was known that autophagy could be modulated in this way, this is the first report detailing the combined effects of CQ and Rapa on chemosensitization. We have also translated this idea into the clinic and were encouraged by the reversal of drug resistance in some patients [26].

## MATERIALS AND METHODS

### Cell culture

Hepatoma cell lines HA22T and Huh7.5.1 were maintained in DMEM (Invitrogen, Verviers, Belgium) containing 10% heat-inactivated fetal bovine serum (FBS), 2 mM L-glutamine, 100 units/mL penicillin, and 100 μg/mL streptomycin (Sigma, St. Louis, MO). Huh7.5.1 is a well differentiated hepatocyte derived cellular carcinoma cell line that was originally taken from a liver tumor in a 57-year-old Japanese male [58]. HA22T is a poorly differentiated hepatoma cell line that was established from hepatoma of a 50- to 60-year-old Chinese male [59].

### Drugs and chemicals

Cisplatin, 5-fluorouracil (5-FU), vinorelbine, docetaxel, and gemcitabine were obtained from Sigma and dissolved in dimethyl sulfoxide (DMSO). Cell culture reagents and consumables were obtained from GIBCO (Gaithersburg, MD) or Corning (Corning, NY). All chemicals not otherwise specified were of the highest grade and were purchased from local suppliers.

### Cell proliferation assay

Hepatoma cell lines were cultured at a density of 1 × 10^5^ cells/well in 96-well round-bottom plates (Falcon, UK) containing 200 μL of medium. Tumor cells (1 × 10^6^ cells) were cultured with various concentrations of chemotherapeutic drugs and CQ, in combination with Rapa, at different molar ratios. Tumor cells were maintained for two days at 37°C in a humidified, 5% CO_2_ atmosphere. The proliferation rate of the cells was measured using an MTS assay (CellTiter 96 aqueous one-solution cell proliferation assay; Promega, WI, USA). 40 μL of CellTiter 96 aqueous one-solution was added to each well. After 4 h of incubation, the UV absorbance of the solution was measured at a wavelength of 490 nm. All MTS assays were performed in triplicate.

### Synergistic analysis

To evaluate the synergism between CQ, in combination with Rapa, and various chemotherapeutic drugs, the results from the cytotoxicity assays were analyzed with CalcuSyn (Biosoft, Cambridge, UK) using the median-effect method, which is a well-established procedure to quantitatively determine whether drug combinations produce greater effects together than expected from the simple summation of their individual effects [60]. The combination index (CI) values obtained from the data reflect the nature of the interaction between different combinations; that is, values < 1 reflect synergistic activity, values = 1 reflect additive activity, and values > 1 reflect antagonism.

### Apoptosis assay

Hepatoma cell lines were cultured and trypsinized, as described above, and washed twice with phosphate-buffered saline (PBS). Apoptosis was assayed using an Annexin V Apoptosis Kit (BD Pharmingen, CA, USA) according to the manufacturer's instructions. Briefly, tumor cells were washed three times with PBS, and then cells were analyzed immediately for apoptosis using Annexin V/PI (propidium iodide) staining. Washed cells were supplemented with 1% BSA and then stained directly with 10 μL of PI and 2.5 μL Annexin V-FITC, after the addition of 222.5 μL of binding buffer. Immediately following a 10 min incubation in the dark on ice, the cells were analyzed by flow cytometry. The percentage of positive cells was determined using a FACSCalibur cytometer and Cell Quest Pro software (Becton Dickinson, Mountain View, CA).

### Acidic vesicular organelles (AVOs) analysis

Huh7.5.1 and HA22T cells were collected in FACS tubes (BD Biosciences Discovery Labware, MA, USA) and resuspended in PBS. The cell suspension was stained with acridine orange (5 μg/ml) for 15 min at room temperature (RT). The cells were washed twice with PBS, resuspended in PBS, and analyzed using flow cytometry and Cell-Quest software. The experiment was performed three times.

### Western blots

Cells were lysed for 5 min at RT in a buffer composed of 150 mM NaCl, 50 mM Tris (pH 8.0), 5 mM EDTA, 1% (v/v) Nonidet p-40, 1 mM phenylmethylsulfonyl fluoride, 20 μg/mL aprotinin, and 25 μg/mL leupeptin (Sigma). The total protein concentration of lysates was measured using the Bio-Rad protein assay (Bio-Rad, Hercules, CA). Cell lysate (100 μg) was electrophoresed on a 12% polyacrylamide gel and the proteins were transferred to an Immobilon-P PVDF membrane (Millipore, Bedford, MA), which was then blocked for 2 h at RT in PBS containing 0.05% Tween 20 and 10% nonfat milk. The membrane was then incubated with antibodies against GAPDH (Sigma), HIF-1alpha, LC3 (Novus Biologicals Inc., Littleton, CO), c-MYC, SQSTM1/p62, PARP, phospho-mTOR, phospho-Akt (Ser 473), phospho-p70S6K, GRP78, phospo-ERK1/2, phospho-PERK, CHOP antibody, (Cell Signaling Technology, Beverly, CA), Glutamine synthetase (GS) (Abcam Inc., Cambridge, MA, USA) for 2 h at RT in PBS containing 0.05% Tween 20 and 5% nonfat milk, followed by incubation for 1 h at RT with horseradish peroxidase-conjugated secondary antibodies (Jackson ImmunoResearch Laboratories, West Grove, PA) in the same buffer. Blots were developed using a chemiluminescent detection system (ECL; GE Life Science, Buckinghamshire, UK).

### Glucose uptake assay

For glucose uptake, 1.5 × 10^5^ cells were stained with the fluorescent D-glucose analogue 2-(N-(7-nitrobenz-2-oxa-1, 3-diazol-4-yl)amino)-2 -deoxyglucose (2-NBDG; 20 μM; Invitrogen) for 1 h, washed with PBS, and analyzed by using a FACSCalibur cytometer and Cell Quest Pro software.

### Phospholipid lipase D activity assay

PLD activity was measured using a PLD activity colorimetric assay kit (BioVision) according to the manufacturer's instructions. Briefly, 5 × 10^5^ cells were washed with PBS and were lysed in PLD Assay Buffer. The samples or standard (50 μL per well) and reaction mix (50 μL per well) were each added to a 96-well plate. Absorbance was measured at 570 nm using a Multiskan FC microplate photometer in a kinetic mode to calculate the PLD activity of the samples.

### Intracellular glutamate assay

Intracellular glutamate concentrations were measured using a Glutamate Assay Kit (BioVision) according to the manufacturer's instructions. Briefly, 1 × 10^6^ cells were washed with PBS and lysed in glutamate assay buffer. The samples or standard (50 μL per well) and reaction mix (50 μL per well) were each added to a 96-well plate. After 30 min incubation, absorbance was measured at 450 nm using a microplate spectrophotometer (Multiskan FC, Thermo Scientific, MA, USA).

### Intracellular ATP assay

The intracellular ATP concentrations were measured using an ATP colorimetric/fluorometric Assay Kit (BioVision) according to the manufacturer's instructions. Briefly, 1 × 10^6^ cells were washed with PBS and lysed in ATP assay buffer. The samples or standard (50 μL per well) and reaction mix (50 μL per well) were each added to a 96-well plate. After 30 min incubation, absorbance was measured at 570 nm using a Multiskan FC microplate photometer (Thermo Scientific).

### Determination of mitochondrial membrane potential (MMP)

MMP were measured using a BD MitoScreen kit (JC-1, BD Biosciences) according to the manufacturer's instructions. Briefly, 1 × 10^6^ cells were washed and resuspended in 500 μL of JC-1 working solution. Cells were incubated at 37°C for 15 min. Following staining, cells were washed twice and resuspended in 500 μL of assay buffer. Cells were immediately analyzed using a BD FACSCalibur flow cytometer. Live cells were gated and analyzed.

### Measurement of ROS production

Intracellular ROS was detected by 2′, 7′-dichlorofluorescein diacetate (DCFDA, Sigma). Cells were washed with PBS and incubated with DCFDA (0.25 μM) for 10 min at 37°C. The florescence intensity was detected using FACSCalibur cytometer and Cell Quest Pro software. Untreated cells were used for normalization.

### Huh7.5.1 hepatoma xenograft model

NOD/SCID mice were obtained from the National Laboratory Animal Breeding and Research Center (Taipei, Taiwan) and used at 6 weeks of age. The Huh7.5.1 cells were harvested by trypsinization, suspended in DMEM supplemented with 10% FCS, centrifuged at 250 × g for 10 min, and resuspended in normal saline at a concentration of 5 × 10^7^ cells/mL before subcutaneous implantation into mice. Male NOD/SCID mice, 6-wk-old, were injected with 5 × 10^6^ cells into the right flank using a 27-gauge needle in a 1 mL tuberculin syringe. Approximately 12 d later, when the tumors reached ~4 × 4 mm in diameter, mice were randomly assigned to specific treatment groups. The longest (a) and widest perpendicular (b) tumor diameters were measured at regular intervals using a caliper. Tumor volume was calculated using the formula V = 1/2 a^2^b. All animals were sacrificed on day 35 following treatment. The animal use protocol was reviewed and approved by the Institutional Animal Care and Use Committee (IACUC) in Shin Kong Wu Ho-Su Memorial Hospital.

### Oxygen consumption rate and extracellular acidification rate

Metabolic responses of Huh7.5.1 and HA22T cells were evaluated with an Extracellular Flux Analyzer (XFe24; Seahorse Biosciences, North Billerica, MA) according to the manufacturer's instructions. The Extracellular Flux Analyzer allows for analyzing oxygen consumption and ECARs of a defined number of cells in a defined small volume of culture media in real time and for monitoring their response to drug treatment. In briefly, cells were seeded 5 × 10^4^ in XFe24-well plates and incubated at 37°C in a 5% CO2 humidified atmosphere for overnight, followed by treatment with indicated drug concentrations for 2 h. After 2 h, the mitochondrial respiration was detected by oxygen consumption rate (OCR) and glycolysis was evaluated by extracelluar acidification rate (ECAR) after injecting the following inhibitors of mitochondrial respiration by oligomycin (inhibitor of ATP synthase, 1uM), FCCP (uncoupling factor), antimycin A/rotenone (inhibitor of mitochondrial complex I of the ETC), and 2-deoxyglucose (2-DG; inhibitor of hexokinase). Basal OCR and ECAR were measured, as well as the changes in oxygen consumption caused by the addition of the metabolic inhibitors described above.

### Statistical analyses

All statistical analyses were performed using the statistical software program Prism 4 (GraphPad Software, Inc., La Jolla California USA, www.graphpad.com). The experimental and control groups were compared statistically using an unpaired two-tailed Student's *t* test. Statistical significance was set at P < 0.05 (denoted as *).
